# Factors associated with successful reintegration for male offenders: a systematic narrative review with implicit causal model

**DOI:** 10.1007/s11292-022-09547-5

**Published:** 2022-12-31

**Authors:** Georgina Mathlin, Mark Freestone, Hannah Jones

**Affiliations:** 1 Centre for Psychiatry & Mental Health, Wolfson Institute of Population Health, Queen Mary University of London, London, UK; 2 The Alan Turing Institute, London, UK

**Keywords:** Directed acyclic graphs, Male offenders, Reintegration, Systematic review

## Abstract

**Objectives:**

This systematic review explored factors associated with successful reintegration into the community for male offenders and investigated which factors may be causally related to reintegration.

**Methods:**

Database searches were conducted in November 2021; a narrative synthesis and associated causal model with directed acyclic graph (DAG) was used to analyse the factors of reintegration.

**Results:**

Thirty-four studies met the inclusion criteria. Risk-Need-Responsivity–based interventions had the strongest evidence for reducing post-release offending. Fourteen good-quality studies met the inclusion criteria. The DAG shows six exposure variables (prison visits, witnessing victimisation, recovery perception, risk assessment, in-prison treatment, and pre-prison health) which link to several post-release outcomes (criminal justice outcomes, drug use, mental health, housing, and reintegration barriers) and confounding variables (demographics, offending history, prior reintegration barriers, substance misuse and attitudes).

**Conclusions:**

The review identified factors that may be causally related to reintegration for male offenders and warrant further empirical investigation.

## Introduction

The end of a prison sentence should be an opportunity for a person to reintegrate into their community and live a prosocial life. However, there is a high likelihood that following release from a prison sentence, a person will reoffend and therefore be returned to prison. In the UK, 23.9% of all released adults reoffend within a year of release, increasing to 58.4% of adults on a 12-month or less sentence (MoJ, 2022). Historical convictions are a known predictor of re-offending and reimprisonment, with evidence suggesting contact with the prison system does not reduce recidivism (Cullen et al., 2011; May et al., 2008). On average, people formerly imprisoned will commit four reoffences, with the social and economic cost of reoffending in the UK estimated to be £18 billion (Newton et al., 2019). Successfully reintegrating people back into the community is therefore an important area for investigation to reduce harm to society and the individuals serving prison sentences. This review therefore asks: what factors are associated with successful reintegration for male offenders? And which of these factors may be causally related to successful reintegration?

Reintegration describes the experience of re-entering the community following a prison sentence, whereas desistance theories attempt to explain why people stop offending. For example, the uniformity aging process (Gottfredson & Hirschi, 1990) suggests people naturally stop engaging in offending behaviour as a result of aging and maturation. However, this theory has been criticised for being simplistic and misinterpreting aggregated data (Weaver, 2019). Alternatively, informal social control (such as education, family, and employment) has been proposed as a way that people may adopt pro-social roles and subsequently cease offending (Laub & Sampson, 1993; Sampson & Laub, 2003). Building on the influence that social control may have on desistance, there are cognitive explanations which suggest alongside informal social control, people need cognitive openness for change to occur (Farrall & Maruna, 2004). Rather than focussing solely on external social factors which may influence desistance, cognitive explanations consider the internal processes that may come prior to or change with the exposure to pro-social bonds (Burnett, 2004). Furthermore, recent theories focus on identity, indicating emotional transformation and social learning are required for desistance (Giordano et al., 2002, 2007). Identity change and decisions to transform are seen as necessary for successful reintegration (Paternoster & Bushway, 2009). Recent theories of desistence focus on individual-level factors which may precede desistence but do not provide adequate explanations for how identity changes occur. The cognitive theories of desistence also ignore interpersonal and structural factors (such as policies, service and housing availability, the prison environment) which may also influence the ability to desist from crime.

Empirical research has focussed on predictors of desistence, falling into four broad areas: the prison sentence, release planning, experience in the community, and attitudes (Dickson & Polaschek, 2014; Folk et al., 2016; Laub & Sampson, 1993; McMurran & Theodosi, 2007). Treatment and offender behaviour programmes during a prison sentence are designed to rehabilitate and reduce reoffending. There is evidence that treatment reduces reoffending (Beaudry et al., 2021; Hanson et al., 2009; Kroner & Yessine, 2013); however, it is important treatment is completed (McMurran & Theodosi, 2007). Alongside treatment, release planning is important. Avoidance-orientated release plans (e.g. do not see *x* person) have been assessed as higher quality (Dickson et al., 2013) and associated with reduced likelihood of reconviction (Dickson & Polaschek, 2014). The quality of release plan has been found to have greater predictive accuracy on recidivism and reimprisonment than dynamic and static risk assessments. Once released into the community, research has shown a person’s connection to their community is related to recidivism (Folk et al., 2016) and that positive social bonds promote desistence (Kay, 2022; Laub & Sampson, 1993, 2001; Walker et al., 2013). The expectations a person has when they are released from prison are also important. LeBel et al. (2008) conducted a 10-year follow-up and found pre-release attitudes were predictive of further offending, indicating a relationship between belief of ability to desist and subsequent offending behaviour (Burnett & Maruna, 2006). Despite there being a range of factors linked to reduced reoffending, the underlying mechanisms of how people successfully reintegrate into the community have not yet been studied together to understand their influence upon one another and provide a more unified understanding of the complex process of reintegration.

Furthermore, research overwhelmingly focuses on recidivism as a sole indicator of successful reintegration, with an emphasis on factors about an individual that may predict recidivism (Barrenger et al., 2021; Visher & Travis, 2003). However, interpersonal- and structural-level factors such as securing housing, gaining employment (Pleace & Minton, 2009), and community aftercare focussing on maintaining therapeutic gains from in-prison therapeutic treatment (Beaudry et al., 2021) may also influence successful reintegration. Post-release outcomes are likely to interact with and influence one another (Wong, 2019) and require consideration together to gain a better insight into the process of successful reintegration. It is therefore necessary to consider a broad range of post-release outcomes alongside reoffending to better understand the mechanisms of the reintegration process. This review aims to examine research exploring individual-, interpersonal-, or structural-level factors related to reintegration with a broad lens to consider their impact on a variety of post-release outcomes. By considering a broad range of research with varying factors and outcomes related to successful reintegration, the complex process of reintegration can be mapped out to distinguish possible causal relationships for further exploration (Ward et al., 2022).

### Objectives

This systematic review asks: what factors are associated with successful reintegration for male offenders? A secondary question was: which of these factors may be causally related to successful reintegration?

The objectives of the review include identifying longitudinal research that investigates factors related to reintegration and to synthesise this research narratively using directed acyclic graphs (DAGs). DAGs are an underutilised tool within criminal justice research. DAGs propose causal relationships between variables (Tennant et al., 2019) and are a useful tool for understanding complex interactions and guiding subsequent research (Elwert, 2013).

## Methods

This review followed the PRISMA 2020 statement checklist (Page et al., 2021; Appendix 4 Table 3). As a meta-analysis was not conducted, an additional checklist of Synthesis Without Meta-Analysis (SWiM) was used as an extension of the PRISMA checklist (Campbell et al., 2020). A protocol was created prior to the review to ensure the rationale and planned methods of the review were pre-specified (Moher et al., 2015).

### Search strategy

Seven databases were searched on 12 November 2021 for relevant literature, these included PsycINFO (via EBSCO), Medline (via OVID), EMBASE, and Web of Science. Grey literature was searched through OpenGrey, PsycEXTRA, and PsycARTICLES. Search terms were created in relation from an extrapolation of the research question to cover the widest range of research exploring the reintegration experience: “Offender*” OR “criminal*” OR “prisoner*” OR “felon*” OR “inmate*” AND “Prison” OR “jail” OR “detention” OR “incarceration” OR “imprisonment” OR “Open prison” OR “Category D” OR “Cat D” OR “correctional home” AND “Progression” OR “release” OR “re-entry” OR “desistance” OR “recidivism” OR “transition” OR “reintegration”. No date restrictions were applied but a filter of English-only papers was applied to each database. No country restrictions were applied to capture the variety of research conducted across geographical regions. 

### Inclusion and exclusion criteria

Table 1 shows the inclusion and exclusion criteria used for the review. The population of interest in the systematic review were adult males serving a prison sentence in any security category. Male-only populations were used as offending trajectories for females are often less persistent (Fergusson & Horwood, 2002) and may have different mechanisms (Cauffman et al., 2015). People in prison transferred to hospital for treatment were not included, as the focus of the review was on reintegrating from a prison setting only. The factor could be individual (age, socioeconomic status, ethnicity, health, attitudes), interpersonal (treatment, prison visits), or structural (prison environment, policies, service availability) variables associated with reintegrating into the community. The outcome was any post-release outcome such as recidivism, securing housing, being in employment, engaging in services, or having healthy relationships. Studies had to be longitudinal but could be qualitative, cohort, quasi-experimental, or RCT. This was to help understand the causality of successful reintegration which occurs over time which cross-sectional or case study designs would not aid.

**Table 1:** Population, factor, outcome, study design inclusion/exclusion criteria

PFO(s)	Inclusion	Exclusion
Population	People serving a prison sentence (in any security category and in prison setting), adult (18 +), male	Any hospital order prisoners
Factor	Any factor associated with reintegration	
Outcome	Any outcome experienced following a progressive move	
Study design	Longitudinal studies published in English	

The included studies had two additional inclusion criteria applied to qualify to be in the DAG development. Firstly, the design was limited to cohort, quasi-experimental, or randomized. Secondly, the papers had to be assessed as “good” quality in the quality assessment. These additional criteria were applied to increase the reliability and trustworthiness of the DAG development.

### Study selection and data extraction

Search terms were entered into the databases and references and abstracts were imported into Rayyan QCRI (Ouzzani et al., 2016), a systematic review online software for screening studies. Duplicate papers were removed at this point. Titles and abstracts were then screened in relation to the inclusion and exclusion criteria by one reviewer (GM). Any unclear decisions were checked with a second reviewer (HJ) and papers not meeting the inclusion criteria were excluded. The remaining papers were read in full by two reviewers (GM and HJ) and only studies meeting the inclusion criteria were then included in the final review. There were no disagreements between the reviewers regarding the inclusion of studies. Data extraction was completed by one author (GM) and a study characteristics table was created for the final papers providing summary details of each paper. Information included date of publication, country, research design, sample characteristics, factor of progression, outcome measures, length of follow-up, and key findings (*p*-values and effect sizes).

### Quality assessment

There were a range of study designs accepted in the review meaning multiple quality assessment tools were used. For qualitative studies, the CASP qualitative checklist (CASP, 2022) was used with the best-quality papers scoring over 17 out of 20. For the quantitative studies, the appropriate National Heart Lung and Blood Institute (NHLBI, 2021) quality assessment checklist was used and rated good, fair, or poor. The NHLBI tool was chosen as it covers broad aspects of quality that would be relevant to assess the included research, including assessing matching in quasi-experimental and controlled trial studies. Quality assessment was completed by one reviewer (GM).

### Synthesis of research

A narrative synthesis of all the included papers was conducted. A meta-analysis was not appropriate due to the heterogeneity of the included study designs and outcomes of included studies meaning no two studies could be meaningfully combined in a meta-analysis. The narrative synthesis involved grouping the studies by factor-outcome relationships and textually writing about the themes and findings of the papers (Popay et al., 2006). Studies were grouped by outcome first (e.g. all studies reporting a reoffending outcome). The factors associated with the outcome (e.g. treatment, family support) were then explored in turn. As an aim of the study was to investigate multiple outcomes, there were many factor-outcome relationships identified across the studies. Only relationships identified in three or more studies are reported to avoid reporting on relationships where there is insufficient data to synthesise.

To consider potential causal factors and pathways to successful reintegration, a subset of included studies assessed as “good” quality and cohort, quasi-experimental, or randomised design were analysed using the Evidence Synthesis for Constructing Directed Acyclic Graphs (ESC-DAG) method (Ferguson et al., 2020). A full description of the ESC-DAG method is provided in Ferguson et al. (2020). Broadly, the ESC-DAG method involves three stages: (i) the mapping of individual study findings into a DAG framework; (ii) assessing the possible causal structure of the DAG using causal inference principles; and (iii) synthesising an “integrated DAG (I-DAG)” from the individual study DAGs (Appendix 1). Causal inference principles in this case emphasise the following: (i) temporal precedence of factor and outcome; (ii) plausibility; (iii) theoretical support; and (iv) a counterfactual thought experiment (“what if the exposure was set to fully present or fully absent”) (Pearl, 2009). DAGitty online software (Textor et al., 2011) was used to develop DAGs in the study.

## Results

### Search results

**Fig. 1:**
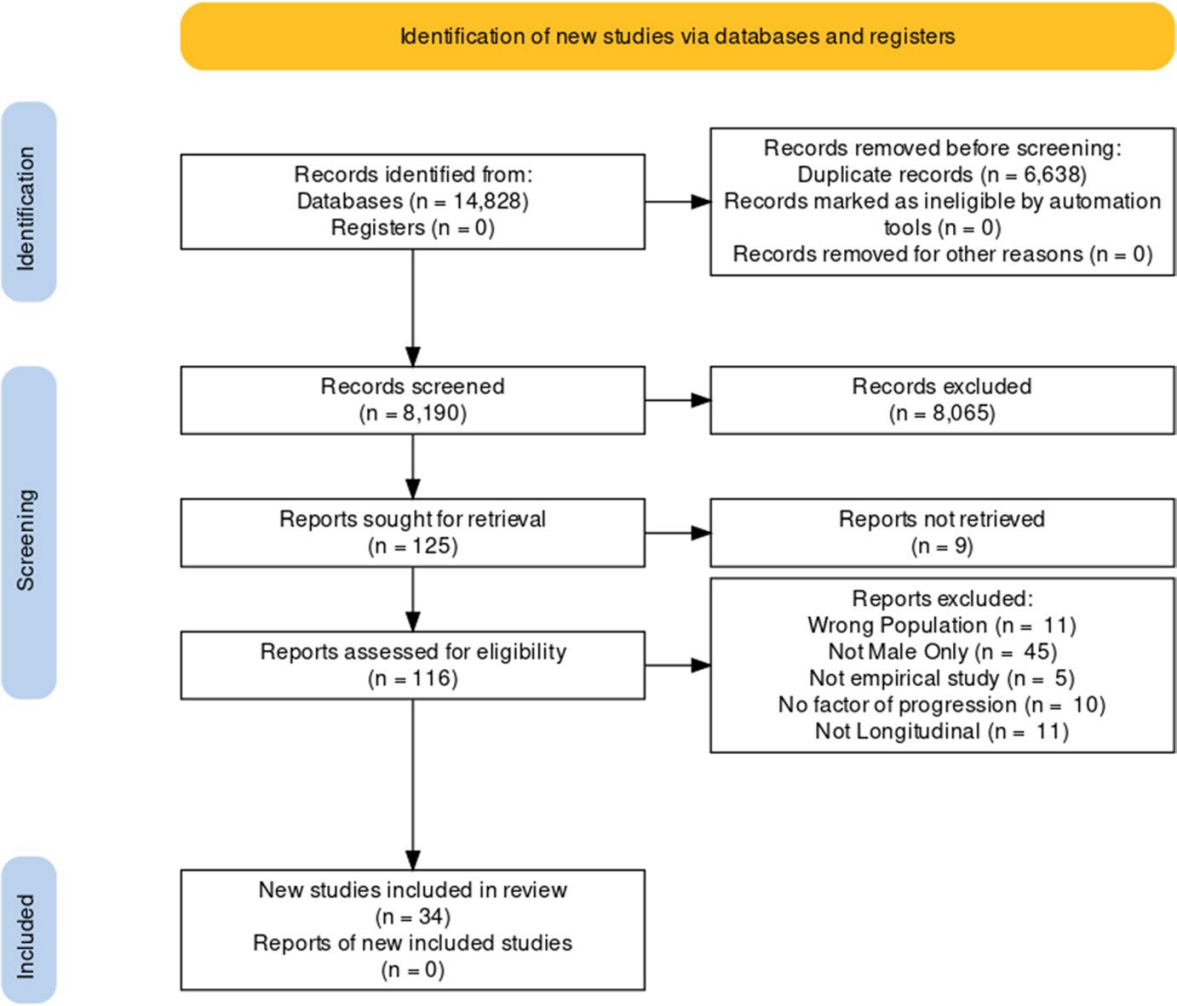
PRISMA 2020 flow diagram

The search terms were entered into each database and a total of 14,828 papers were retrieved, decreasing to 8190 unique entries once duplicate papers were removed. Search results were screened using Rayyan QCRI (Ouzzani et al., 2016). The 8190 studies titles and abstracts were screened for relevance which led to 116 full-text papers being assessed for eligibility. Thirty-four papers met the inclusion criteria and were included in the narrative synthesis. With the additional inclusion criteria applied, 14 papers were included for the I-DAG construction (Fig. 1).

### Study characteristics

The key characteristics of each study contributing to the narrative synthesis and DAG construction can be found in Appendix 3 Table 2. Most studies were conducted in the USA (*n* = 23), with cohort studies the most common design used (*n* = 17). The studies were all longitudinal. The length of follow-up ranged from 1 month to 15 years (mean = 2.34 years, median = 2 years). All samples consisted of males over the age of 18. The average age of the included study samples was 33 (3 studies did not provide information about age). Sentence lengths ranged from 6 months to 6.5 years with 37 months (3 years) the average sentence length across the samples. Thirteen studies did not report the sentence length of their participants. The samples mainly had a high proportion (56%) of Black/African American ethnicity populations. Six studies did not provide information about the ethnicity of their sample. Only 5 studies provided information about the mental health status of their sample, and these were all reported in varying ways.

The studies reported a range of individual, interpersonal, and structural factors and outcomes related to reintegration: Individual factors (*n* = 11) included health, self-esteem, religiosity, and attitudes. Interpersonal factors (*n* = 28) reported were engagement with treatment programmes and family/social support. The experience of the prison environment was the only structural factor recorded across the studies (*n* = 4). Post-release offending (*n* = 27), attitudes (*n* = 4), drug use (*n* = 5), and mental health (*n* = 3) were individual-level outcomes reported across the studies. Employment (*n* = 8) was the only interpersonal-level outcome reported and the experience of reintegration (*n* = 2) was the only structural-level outcome explored.

### Outcomes

#### Post-release offending behaviour

##### In-prison treatment

Four studies reported on cognitive-behavioural (CBT) type interventions including reasoning and rehabilitation, violence reduction programme, and criminal attitudes programme (Baggio et al., 2020; Berman, 2004; O'Brien & Daffern, 2017; Simourd et al., 2016). These studies reported reduced likelihood of post-release offending, mediated through attitude change and treatment completion, with unclear results on the lasting impact of the treatment. These studies were assessed as “fair” quality.

Five studies examined Risk-Need-Responsivity (RNR) based treatment (Dockery, 2019; Lattimore & Visher, 2013; Lugo et al., 2019; McNeeley, 2018; Visher et al., 2017). All found a lower likelihood of post-release offending with one study (Lattimore & Visher, 2013) reaching significance in the 3-month follow-up period. RNR-based treatment was especially effective for first-time offenders (Dockery, 2019) and those in the first year of their sentence (Lugo et al., 2019). The evidence for RNR reducing re-offending is strong with consistent findings across high quality studies.

##### Experience of prison

Witnessing others being victimised and poor behaviour during the sentence were predictive of poor offending outcomes across four studies (Daquin et al., 2016; McDougall et al., 2013; Walters, 2016, 2020). The papers present consistent findings and have “good” to “fair” quality.

#### Post-release employment

##### In-prison treatment

Receiving treatment during a prison sentence was associated with increased likelihood of being in employment post-release (Jensen et al., 2020; Jung, 2014; Lattimore & Visher, 2013; Walters, 2020). These studies are good-to-fair quality, so the evidence of in prison treatment improving likelihood of post-release employment is strong.

#### Post-release drug use

##### Support

Improvements in family relations and religious support throughout a prison sentence was significantly associated with reduced likelihood of drug use post-release (Brunton-Smith & McCarthy, 2017; Farrell, 2009; Stansfield et al., 2019). The consistent findings across these good-quality papers provides strong evidence for the impact of family relations and religious support on post-release drug use.

##### In-prison treatment

Re-entry–focussed interventions were associated with reduced likelihood of marijuana use in a 3-month follow-up period (Lattimore & Visher, 2013) and reduced overall substance abuse at 15 months post-release (Stansfield et al., 2019). A mindfulness-based intervention had no effect on post-release substance use (Malouf et al., 2017). This indicates varying evidence for in-prison treatment on post-release drug use, with two “good”-quality studies providing consistent findings and one poor-quality study presenting an opposite conclusion. The type of treatment provided is likely important when considering substance use outcomes.

#### Post-release attitudes

##### In prison treatment

CBT-based and employment-based treatments were associated with positive impacts on post-release attitudes of hope, denial and minimisation, and criminal attitudes (Medlock, 2009; O'Brien & Daffern, 2017; Simourd et al., 2016). The mindfulness-based intervention found no difference in motivation and self-control compared to a control group (Malouf et al., 2017). These studies have fair quality and therefore further high-quality research is needed to understand how in-prison treatment can impact post-release attitudes.

#### Reintegration experience

The reintegration experience describes a range of aspects of a prosocial life for offenders once released into the community including support, housing, and supervision. Poor support, including familial conflict, lack of employment, and financial issues are reported as barriers to reintegration by Link et al. (2019), Russell et al. (2013), and Naser and La Vigne (2006). In a qualitative study, offenders described fearing community reaction, wanting pre-arranged accommodation and an existing relationship with a probation officer when interviewed pre-release and once released reported finding negative reactions stressful, having accommodation issues and unsupportive probation officers (Russell et al., 2013).

### ESC-DAG

Fourteen studies (marked with an * in the study characteristics table) were included in the construction of the I-DAG.

#### I-DAG analysis

**Fig. 2:**
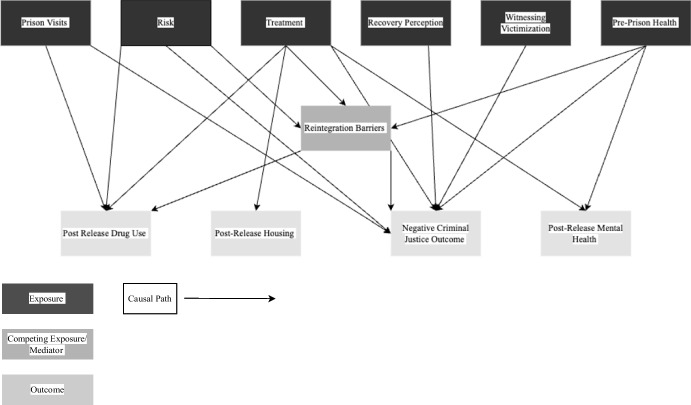
I-DAG of causal paths

Figure 2 shows the causal paths I-DAG which represents the factors of reintegration (exposure variables) and how they relate to the reintegration outcomes. Appendix 2 Fig. 9 shows the full I-DAG which has five types of variables, reflecting the application of causal principles to the DAG:

- Exposure variables are the cause of an outcome and were defined as an exposure in the reviewed studies.
- Outcome variables are the consequence of an exposure variables and were identified as the outcome of interest in the reviewed studies.
- Confounder variables cause both the exposure and the outcome.
- Mediating variables are an effect of the exposure that leads to the outcome.
- Competing exposure variables cause the outcome but have no relation to the focal exposure variable.

A causal (or direct) path is apparent when one variable connects to another with an arrow. The focal relationships in the I-DAG are the paths between exposures (darkest grey) and outcomes (lightest grey) and imply there is a causal effect between the variables. Confounding variables create confounding (indirect or backdoor) paths between an exposure and outcome and introduce confounding bias (dashed arrows). Where a competing exposure is causing an outcome, a dashed arrow is used in the figures.

Figure 2 shows 6 exposure variables (prison visits, witnessing victimization, recovery perception, dynamic risk of reoffending, in-prison treatment, and pre-prison health), 1 mediating variable (barriers to reintegration), and 4 outcomes (post-release drug use, negative criminal justice outcomes, housing, and post-release mental health). To understand the I-DAG further, each exposure variable is considered in isolation.

##### Prison visits

**Fig. 3:**
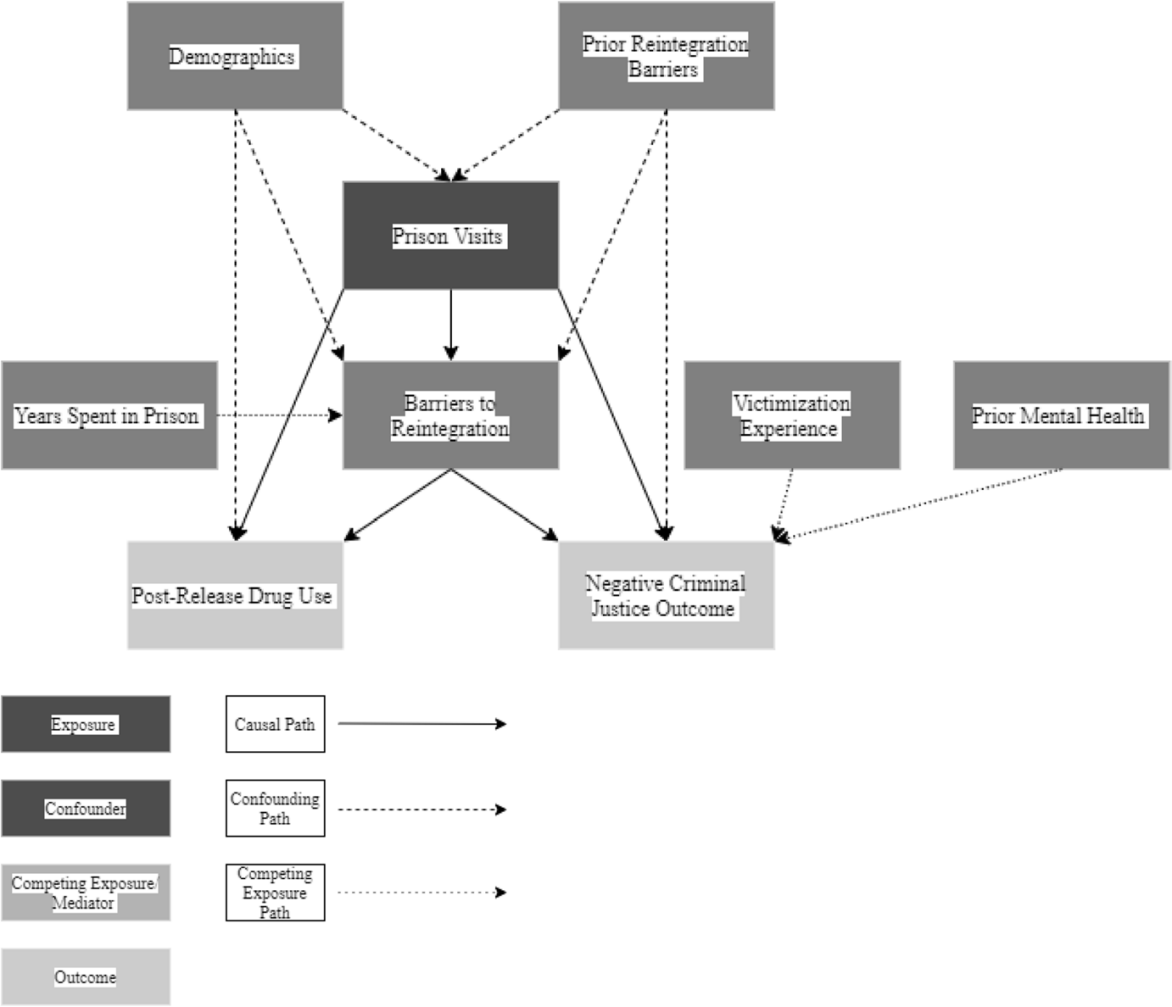
Prison visits DAG

This variable describes if a person received visits during their prison sentence. Following the synthesis of systematic review studies, receiving prison visits was linked to reduced likelihood of post-release drug use and negative criminal justice outcomes, mediated through barriers to reintegration. Prior reintegration barriers and demographics were confounding variables in the relationship between prison visits and both its associated outcomes. There were 5 causal paths showing direct and indirect effects of prison visits on drug use and negative criminal justice outcomes (Fig. 3).

1. Prison visits → Post-release drug use
2. Prison visits → Negative criminal justice outcome
3. Prison visits → Barriers to reintegration
4. Prison visits → Barriers to reintegration → Drug use
5. Prison visits → Barriers to reintegration → Negative criminal justice Outcome

##### Witnessing victimization

**Fig. 4:**
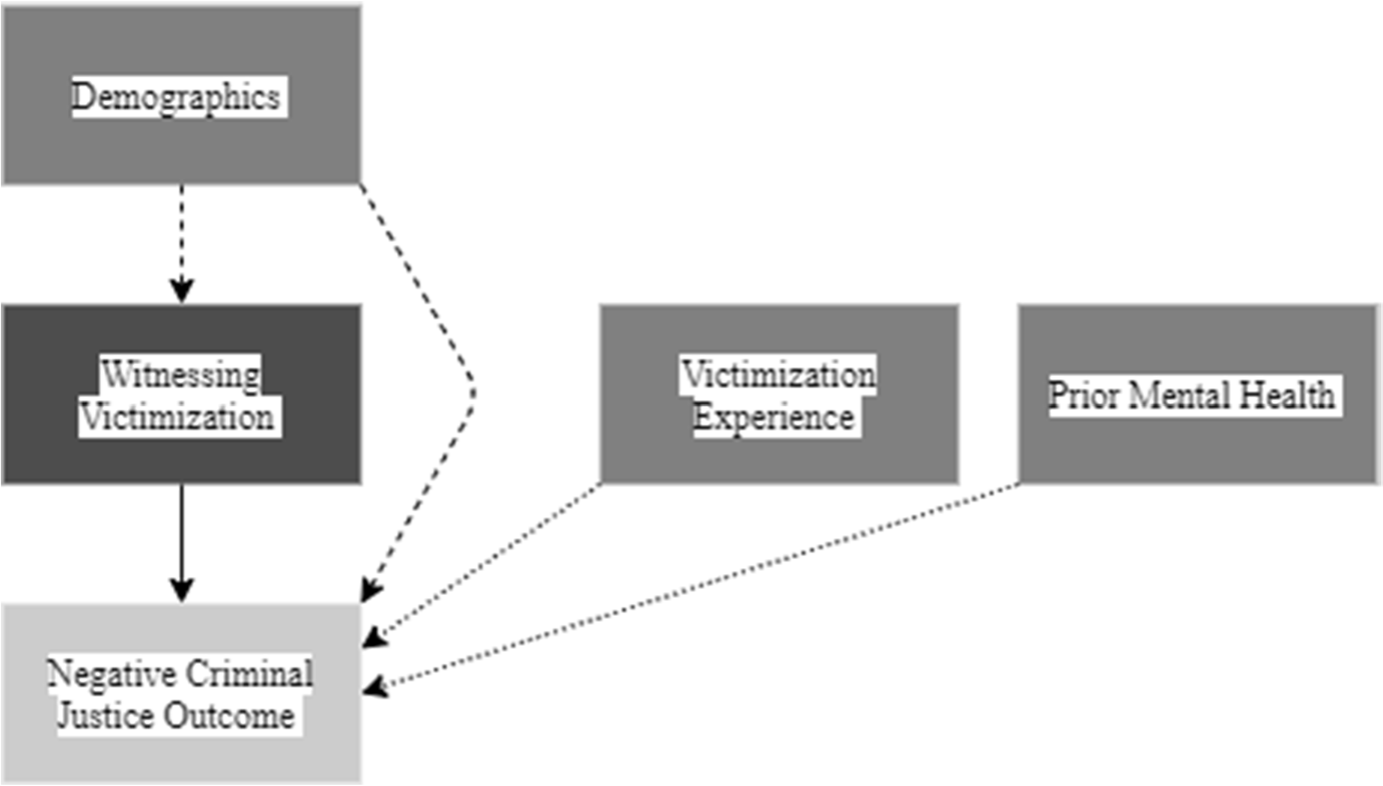
Witnessing victimization DAG

Witnessing victimization describes if the offender observed any abuse, physical or psychological, during their sentence. The DAG shows witnessing victimisation was only related to negative criminal justice outcomes with demographics as a confounding variable in this relationship. There is one direct causal relationship (Fig. 4).

##### Recovery perception

**Fig. 5:**
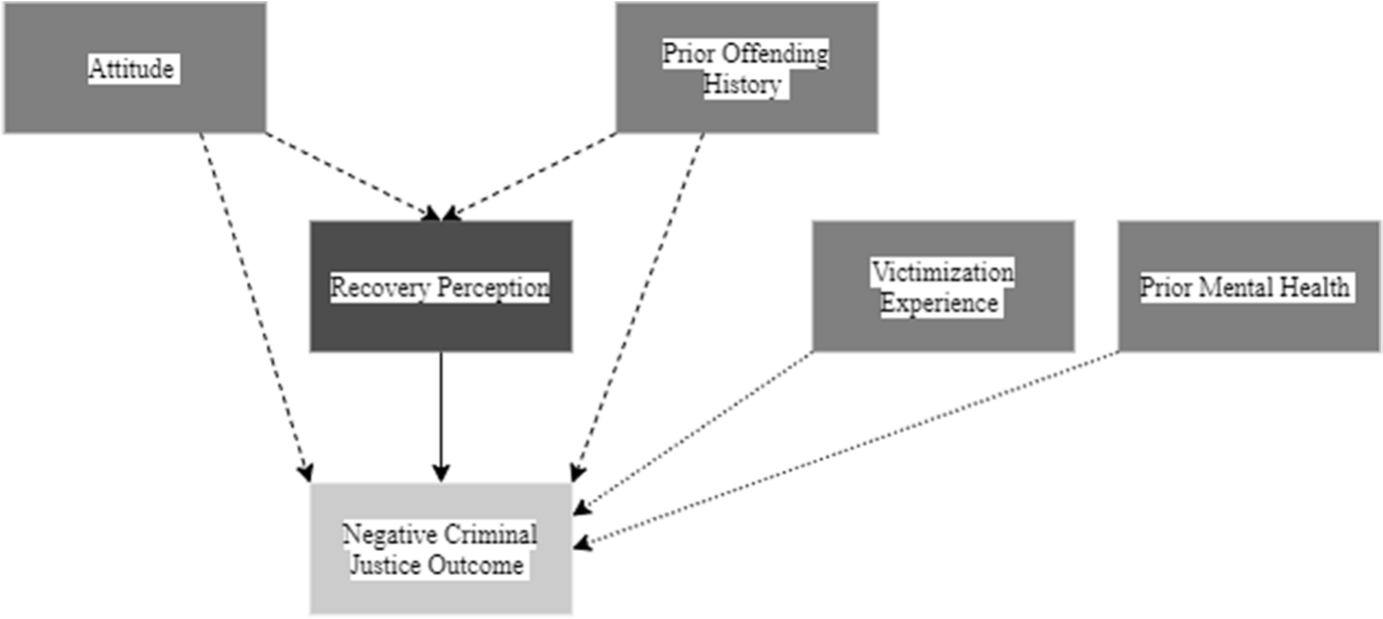
Recovery perception DAG

Recovery perception describes how much the offender feels they have recovered during their sentence. In the DAG, poor recovery perception was related to any negative criminal justice outcome with offending history and attitudes during the prison sentence being confounders in this relationship. There is one direct causal relationship (Fig. 5).

##### Risk of reoffending

**Fig. 6:**
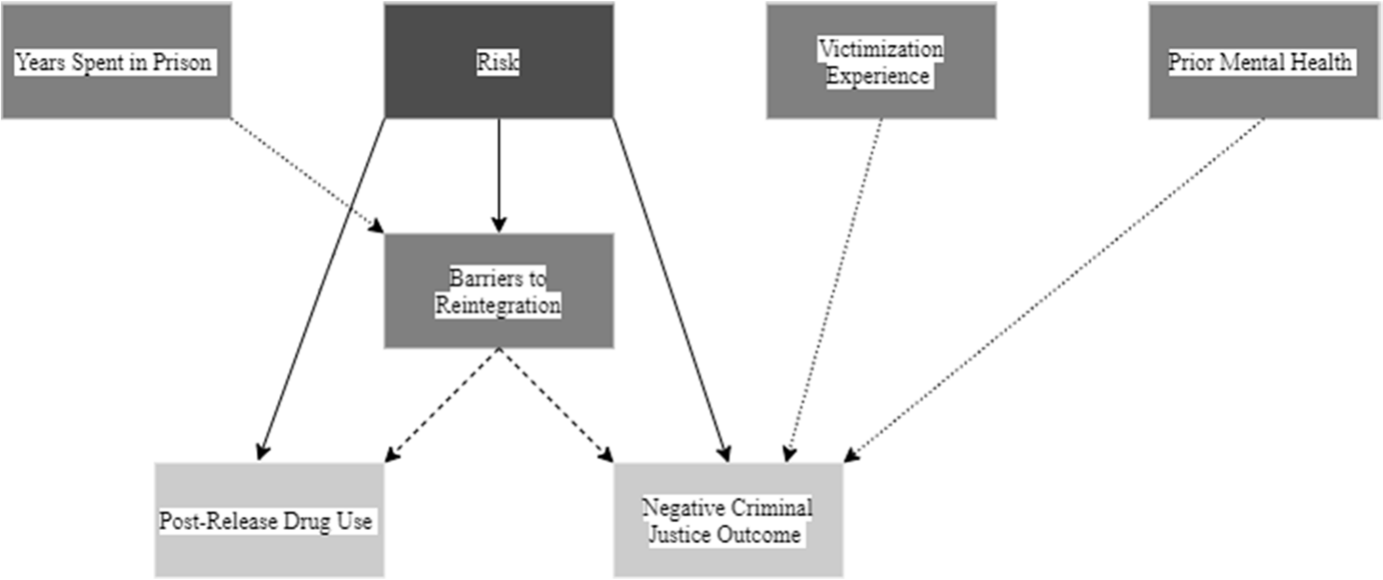
Risk DAG

Risk of reoffending describes a variety of static and dynamic factors related to the likelihood of reoffending (e.g. age at first offence, number of prior offences, current age, employment, having criminal friends, problematic substance use, psychological problems, difficult family relationships, attitudes supportive of crime, and years incarcerated). The risk variable also included a measure of behaviour during the prison sentence (behaviours of “concern” or “positive” behaviours) and was identified as an exposure in the reviewed studies. The DAG shows increased risk of reoffending being related to post-release drug use and negative criminal justice outcomes, mediated through barriers to reintegration. There are therefore two indirect causal relationships related to risk (Fig. 6). There are no confounding variables influencing risk and the outcomes in this model and so it may be better conceptualised as a confounder itself.

1. Risk → Barriers to reintegration → Drug use
2. Risk → Barriers to reintegration → Negative criminal justice outcome

##### Treatment

**Fig. 7:**
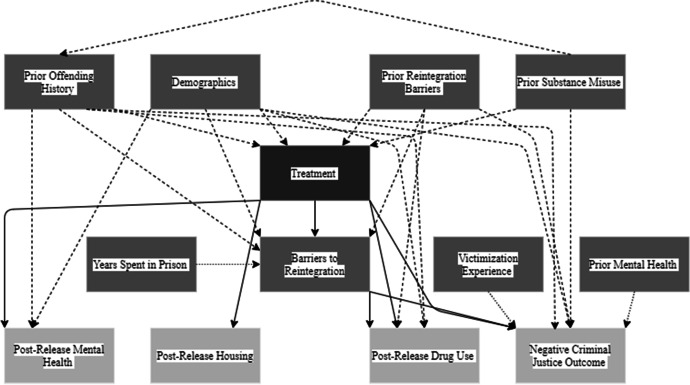
Treatment DAG

Treatment describes the undertaking of any in-prison intervention or programme. The DAG shows treatment being related to reduced post-release drug use, negative criminal justice outcomes, and improved post-release housing and post-release mental health. Prior reintegration barriers, demographics, prior substance abuse, and offending history were confounders and there are four direct and two indirect causal pathways (Fig. 7).

1. Treatment → Drug use
2. Treatment → Negative criminal justice outcome
3. Treatment → Housing
4. Treatment → Post-release mental health
5. Treatment → Barriers to reintegration → Drug use
6. Treatment → Barriers to reintegration → Negative criminal justice outcome

##### Pre-prison health

**Fig. 8:**
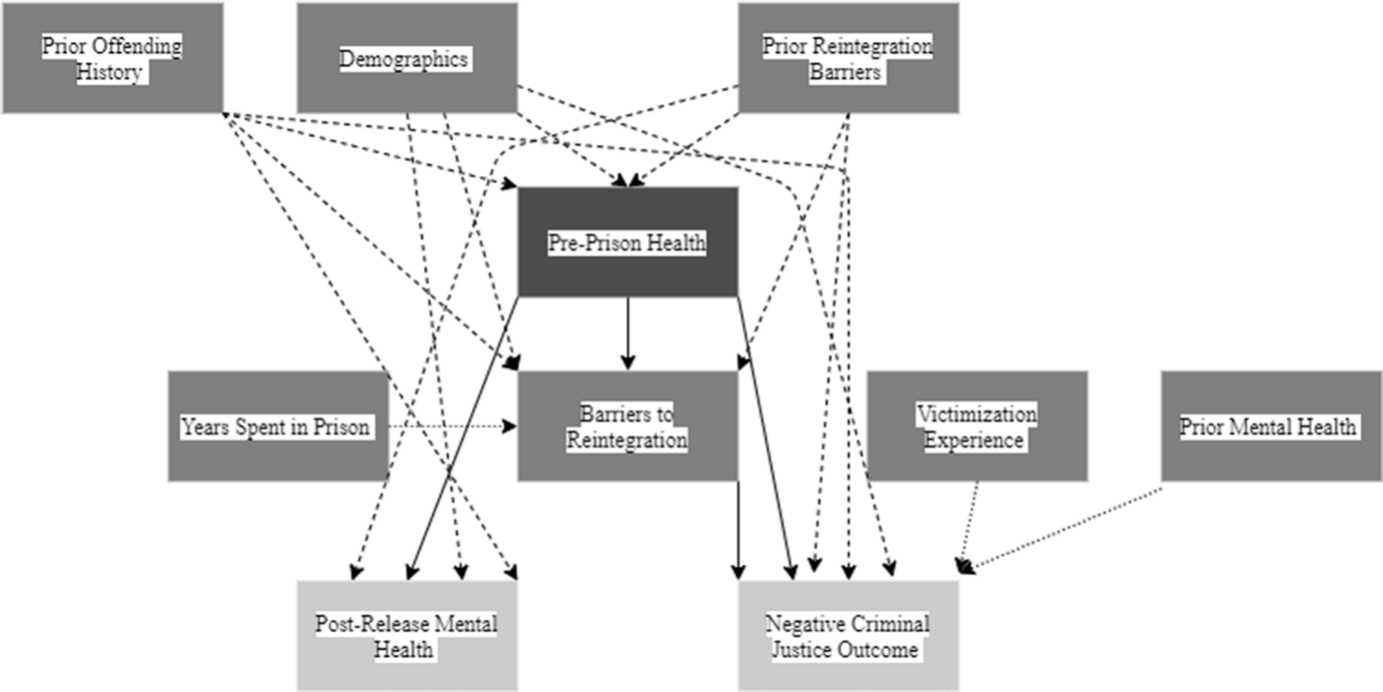
Pre-prison health DAG

**Fig. 9:**
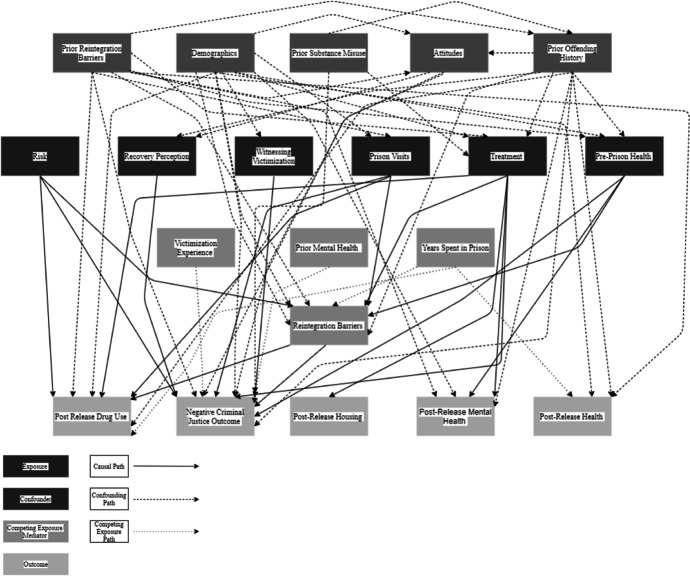
Full I-DAG demonstrating causal, confounding, and competing exposure paths across variables of the 14 studies with key

Pre-prison health describes the physical and mental health of the offender as they enter their prison sentence. The DAG shows pre-prison health is related to negative criminal justice outcomes and post-release mental health and mediated through barriers to reintegration with prior reintegration barriers, demographics, and offending history as confounder variables. There are three direct causal relationships and one indirect causal pathway (Fig. 8).

1. Pre-prison health → Barriers to reintegration
2. Pre-prison health → Negative criminal justice outcome
3. Pre-prison health → Post-release mental health
4. Pre-prison health → Barriers to reintegration → Negative criminal justice outcome

## Discussion

This review aimed to understand what factors are associated with successful reintegration for male offenders and found 34 longitudinal studies investigating factors of reintegration into the community. The narrative synthesis indicated the strongest evidence was presented for RNR-based interventions reducing the likelihood of post-release offending, as well as there being a negative relationship between witnessing victimisation of other offenders during a prison sentence and post-release offending. Further strong evidence was reported for the impact of family and/or religious support on post-release drug use. Moderate support was provided for the relationship between CBT and post-release offending, re-entry services impacting post-release drug use, and CBT and employment-focussed programmes on post-release attitudes. Many studies were assessed as poor or moderate in the quality assessment; and therefore, the reliability of the findings of the narrative review is limited.

The ESC-DAG method was used to develop an understanding of possible casual relationships between dynamic variables and positive progression using the studies assessed as good quality. The I-DAG synthesised relationships from 14 studies and following assessment led to six exposure variables (prison visits, witnessing victimisation, recovery perception, risk of reoffending, in-prison treatment, and pre-prison health) being linked to varying post-release outcomes (including criminal justice outcomes, drug use, physical and mental health, housing, and reintegration barriers) with a variety of confounding variables and one mediating variable (barriers to reintegration) impacting the focal exposure—outcome paths. The synthesis of research evidence using DAGs helps map out the complex relationships of reintegration based on the current literature. There was a small amount of good-quality research with robust study designs found through this review and the conclusions that can be made from the synthesis are therefore limited.

The I-DAG does however set out some possible relationships, some of them causal, between those factors. Firstly, the exposure variables cover interpersonal factors such as receiving treatment, prison visits, and witnessing victimisation and its impact on reoffending, drug use, mental health, and housing. Receiving treatment was linked to the most post-release outcomes including a reduced likelihood of reoffending and post-release drug use, increased likelihood of securing housing, and improved post-release mental health. Recent reviews have found treatment for people in prison requires improvement as the benefits are limited by publication bias and small-study effects (Beaudry et al., 2021; Gannon et al., 2019). There is a lack of understanding of mechanisms of change due to treatments currently used in prisons and there are ethical issues surrounding treatment (e.g., who gets selected for treatment) that may mean minority groups remain over-represented in prison statistics (Ward et al., 2022). To ensure treatment in prisons has a positive effect on post-treatment outcomes, more work is needed to understand the theory and implementation of these treatments, rather than only focussing further on whether treatments are effective (Rogers, 2008).

Prison visits were linked to reduced post-release drug use and reduced likelihood of negative criminal justice outcomes. Reviews into the impact of prison visits on post-release outcomes indicate research is low quality but there are consistent findings on the positive impact visits have on well-being and reduced recidivism (De Claire & Dixon, 2017; Mitchell et al., 2016). Further high-quality research is therefore needed to understand how and why prison visits may lead to positive post-release outcomes. It is also important to understand the impact the lack of prison visits people in prison received during the COVID-19 pandemic may have had. If prison visits are an essential contributing factor to successful reintegration, then the lack of this contact for up to 2 years is likely to have led to detrimental effects on reintegration (Casey et al., 2021).

Witnessing victimisation was only linked to increased likelihood of negative criminal justice outcomes and the effect witnessing victimisation may have on other post-release outcomes is an area for further exploration. A review of exposure to potentially traumatic events (which only included direct victimisation) indicated few poor-quality studies have explored this link but traumatic events during a prison sentence are associated with poorer psychological well-being outcomes post-release (Piper & Berle, 2019). There is a high likelihood of being exposed to a traumatic event or witnessing victimisation during a prison sentence; and therefore, further work is needed to identify how these events can be reduced. Prison culture is an arguably modifiable factor that, if improved, could reduce the likelihood of traumatic events and victimisation occurring and should therefore be the focus of future research and policy (Wooldredge, 2020).

The I-DAG also identifies individual-level factors that can influence post-release outcomes, such as pre-prison health and recovery perception. Individual-level factors often receive research attention as they are viewed as modifiable; however, it is important to acknowledge that factors, such as health, are influenced by interpersonal and structural factors (e.g. geography, socio-economic status; Wan, 2008). The health of a person before their prison sentence was linked to post-release mental health and the likelihood of having a negative criminal justice outcome. Health care has been focussed on as part of the resettlement process, for example RECONNECT in England are services that aim to improve the well-being of prison leavers, reduce inequalities, and address health-related predictors of offending behaviours. These services are yet to be systematically evaluated; however, it is hoped they will increase successful reintegration to the community. There is a lack of intervention and policy focussed on health prior to possible involvement with the criminal justice system and a public health approach to crime is an under-studied area (Akers et al., 2012).

Recovery perception describes attitudes about an individual’s ability to desist from criminal behaviour. According to the I-DAG, people with a poor recovery perception are more likely to have negative criminal justice outcomes. Readiness for treatment and change have been explored through the Multifactor Offender Readiness Model (MORM; (Ward et al., 2004) where individual-level factors (such as attitudes) are required in order to be ready for change. Despite the utility of the MORM in identifying a variety of individual and contextual factors to assess readiness for treatment and change, the model does not provide an explanation of how people develop their beliefs to begin with or how to target negative beliefs, prior to engagement with treatment. It is likely that people have ingrained beliefs regarding their ability to desist, which may be linked to their perception of control over their situation during a prison sentence, but this needs further exploration.

Barriers to reintegration (issues with family, housing, employment, and finances) were a mediating factor in many of the relationships in the I-DAG and explain the link between many of the exposures and outcomes. Barriers to reintegration therefore warrant attention as they could improve post-release outcomes. Release planning is an area where these factors can be focussed on to ensure when people are released, they have positive family and financial support alongside housing and employment options. UK open prison establishments provides access to Release on Temporary License (ROTL) where individuals can develop links in the community, such as family, housing, employment, and support services. People who access ROTL during their prison sentence are less likely to reoffend (Cheliotis, 2008); however, ROTL is often under-utilised in criminal justice systems.

Risk of reoffending was identified through the I-DAG analysis as a factor related to reintegration; however, it differed from the other factors of reintegration as no confounding variables were related to risk of reoffending and the associated post-release outcomes (post-release drug use and negative criminal justice outcome). Risk may therefore be a confounder itself in the broader exposure outcome relationships identified in the I-DAG. Dynamic risk factors have been used as key predictors of reoffending and are the target for many interventions aimed at reducing criminal behaviour (Bonta & Andrews, 2007). To further capitalise on the possibility of dynamic risk factors having a causal association to post-release outcomes, a risk-causality method has been proposed by Heffernan et al. (2019) to aid an individualised formulation for the needs of each person. The method takes a dynamic risk factor and considers possible causes, contexts, and behavioural and mental states to explain how the risk factor may lead to a negative release outcome (Heffernan et al., 2019) for an individual. Further use of the risk-causality method to understand an individual’s dynamic risk may lead to more effective engagement with changing these risk factors and therefore better release outcomes.

### Limitations of the studies included in the review

The quality of the research was mixed, with the quasi-experimental studies being higher quality than the other designs. There were two key limitations of the studies included in the review. Firstly, studies were mainly retrospective observational studies with sample size and power issues. Although this is easier data to collect and analyse in forensic populations, this can lead to underpowered studies which do not add much to the evidence base compared to adequately powered prospective studies which would provide more reliable results. Secondly, the follow-up times across the studies were short (around 2 years on average) which limits the understanding of the reintegration process and outcomes. This is a particular issue as we build evidence to understand reintegration better, which is an ongoing process.

Each exposure variable in the I-DAG was related to criminal justice outcomes and most of the evidence in the review (27 out of 34 studies) focussed on this outcome, showing the continued focus on recall and reoffending in this area of research (Barrenger et al., 2021). Although understanding factors which may be related to reoffending and negative criminal justice outcomes is important, there needs to be an increased focus on broader post-release outcomes and how these relate to negative criminal justice outcomes (Wong, 2019).

### Strengths and limitations of the review

A key strength of this systematic review is the use of the ESC-DAG method to develop a causal synthesis of the included studies beyond the narrative synthesis. Understanding causal mechanisms allows a greater understanding of complex systems (such as a criminal justice system) compared to focussing on isolated predictive factors (Matsueda, 2017) and the I-DAG sets out areas for future investigation and refinement. The I-DAG will also help future researchers identify potential confounding variables and therefore statistically adjust for this in their analysis.

A limitation is that only studies in English were included, and this may have led to the exclusion of relevant research. In the synthesis of the studies, it was decided to group together any criminal justice outcome; however, this meant the relationship between factors of reintegration and specific offending outcomes, such as recidivism or recall, is not understood from this review. Likewise, treatment was grouped into a single variable in the synthesis; and therefore, a nuanced understanding of specific treatment interventions and their impact on specific post-release outcomes is not developed from the review. The I-DAG only represents pathways explored in the included studies and other plausible pathways are not represented. Furthermore, the I-DAG shows no interaction between the post-release outcomes, but it is likely the post-release outcomes are inter-connected (e.g. substance use and lack of housing increasing the risk of recall or reoffending), and future research should seek to explore how outcomes may be related in more detail. However, the robustness of the ESC-DAG methods will have helped synthesise the current research and identify where possible causal relationships exist.

### Future directions

Following the development of the I-DAG, there are several implications for future research and policy. More research is needed into the implementation of prison interventions to ensure effective treatments are being delivered. Further research is needed into how and why prison visits may lead to positive post-release outcomes and the impact that COVID-19 has had on stopping prison visits. There is a need for more understanding of how beliefs about reoffending can be changed. This is an area that would benefit from more research with people who have managed to desist to understand their experiences of desisting.

At a policy level, prison environments should be improved to reduce witnessing and experiencing victimisation and temporary release could be utilised further to help reduce barriers to reintegration. At a broader level, a public health approach to crime reduction could benefit people prior to a prison sentence as well as when reintegrating into the community. It is clear systems and structures impact post-release outcomes, including the socio-political context, and it is important these structural-level factors are included in future research about reintegration. Furthermore, research should seek to understand the interaction across post-release outcomes as this will lead to a greater understanding of the way different factors interact post-release, rather than simply looking at the pre- to post-release relationships. The I-DAG developed in this review provides a basis for which further research can be designed to test the validity of the proposed relationships as well as consider the impact of confounding variables.

## Conclusion

There are many factors related to successful reintegration for male offenders; however, there is a lack of good-quality research with an over-reliance on studying treatment efficacy and reoffending which neglects a wider systemic approach considering individual-, interpersonal-, and structural-level influences of successful reintegration. Developing causal models through DAGs helps understand the complex, multifactored process of reintegration and provides a basis for future research to expand upon and test.

## Data Availability

All data and materials used in the review are available upon request.

## Appendix 1 Summary of ESC-DAG procedure

1. Mapping each study into an “implied graph” which undergoes scrutiny to be translated into a study DAG.
  - The study variables are identified (exposure, outcome, mediators, controls).
  - A directed edge is drawn from the exposure(s) to the outcome(s).
  - Controls are unassigned variables and a directed edge is drawn from each control to the exposure(s) and outcome(s).
  - Mediators are added in as the study identifies.
  - Some recombination may be needed to simplify a complex implied graph. For example, age, sex, and marital status variables may be combined into a demographic variable.
2. Translation of each implied graph into a DAG for each study. All relationships in the implied graph are assessed through the following causal criteria (firstly in the directed edge direction and then the reverse).
  - Temporality—does the cause proceed the effect?
  - Face-validity—is it plausible?
  - Recourse to theory—is there evidence for the relationship?
  - Counterfactual thought experiment—using the potential outcomes framework (POF) compare the outcome that would have occurred if all the sample had been exposed compared to the outcome if none of the sample had been exposed.
  - Once all these criteria have been assessed for the relationship, the edge is either retained, reversed, or deleted.
  - Once all relationships in the implied graph have been assessed, a DAG of the study is created, and the directed edges are recorded into an index.
3. Integrating the DAGs takes two stages. Firstly, the translated DAGs are combined into one using the indexed directed edges.
  - Starting with the exposures and outcomes, the nodes are added to the I-DAG.
  - The confounding nodes are then added, and all directed edges inputted.
  - Conceptually similar nodes can be grouped together in the virtual space, ready for recombination.

Secondly, the I-DAG is recombined into a conceptual I-DAG. This is to reduce the complexity of the I-DAG. The two reasons for combining nodes are as follows:

- Nodes can be combined if there is theoretical support (e.g. demographics and sociodemographic).
- If the conceptually related notes have similar inputs and outcomes (e.g. do they have directed edges going to similar places in the I-DAG?).

## Appendix 2

Figure 9

## Appendix 3

Table 2

**Table 2:** Table of characteristics and quality assessment

Authors, date of publication, country	Design	Sample	Factor(s) of Progression	Outcome measure(s)	Length of follow-up	Key findings	Quality assessment (NHLBI)
Baggio et al., 2020 Switzerland	Quasi-experimental	*N* = 213 Intervention (*N* = 139, 21 dropouts) Age = 34.07 TAU (*N* = 84, 25 dropouts) Age = 35.71	Reasoning & Rehabilitation Programme 14 × 90 min sessions	Recidivism (new offence) Inventory of Interpersonal Problems (IIP) Aggressiveness Hostile cognitive distortions Willingness to accept responsibility	4.5 months	21.6% (*N* = 11) of released (*N* = 51)) reoffended Group assignment had a marginal effect on recidivism (OR = 0.75, *p* = 0.06) R&R group significantly less spontaneous aggression (*p* = 0.04, MD = 0.21)	Fair
Berman, 2004 Sweden	Quasi-experimental	Intervention: *N* = 339 (256 completers, 83 dropouts) Control: *N* = 550 (430 matched completers, 140 match dropouts)	Social Learning Theory–based Reasoning and Rehabilitation Programme 36 2-h sessions	Reconviction (1 > court adjudications, resulting in a new sentence to prison or probation)	3 years	Significant effect of R&R programme at 36 months (*p* < 0.01) Programme completers 25% less likely to be convicted than controls (p < 0.02). Dropouts 38% more likely to be convicted than controls (p < 0.05) Effects after 36 months not found Programme completers with medium risk (p < 0.05), prior violent convictions (p < 0.05) and aged 31–44 (p < 0.05) showed lower reconviction rates	Fair
*Brunton-Smith et al., 2017 UK	Cohort study	*N* = 2617 Sentence length: 6 months – 4 years Interviewed on reception into prison, 2 weeks prior to release, and 2 months post-release	Family attachment (self-report) Family prison visits (self-report)	Proven reoffending – PNC Employment (self-reported) Class A drug use (self-reported)	2 years	Significantly lower levels of reoffending for improving family relations across their sentence (*p* < 0.001) Improvements in family relations associated with significantly lower drug use (*p* < 0.01) and more employment (*p* < 0.05) in the month following release No influence of type of family visit received	Good
*Daquin et al., 2016 Ohio, USA	Cohort study	*N* = 1613 Age: 18–77 years old (*M* = 34) Ethnicity: 46% white Marital status: 89% married Prior prison: 53% Religious: 54% Mental illness: 18%	Victimisation experiences (witnessing verbal assaults, fighting, theft or sexual) in last 12 months of most recent incarceration (self-report)	Arrest (parole officer case notes/online record checks) Any negative criminal justice outcome: arrest, parole violation, revocation, reincarceration	2.5 years	Witnessing theft increased odds of any negative criminal justice outcome at 85% higher rate (OR = 1.85, 95% CI) Witnessing sexual victimisation increased chances of parole violation by 35% (OR = 1.35, 95% CI) Witnessing sexual victimisation increased chances of rearrest by 44% (OR = 1.44, CI 95%) Age (older) lowered odds (OR = 98, CI = 95%) and mental illness increased odds (OR 1.49 CI = 95%) of being arrested	Good
*De Leon, Melnick, Cao, and Wexler, 2006 San Diego, USA	Cohort study	*N* = 526 (313 treatment, 213 control), 268 for 3-year prediction analysis (196 treatment, 72 control) Age: *M* = 31.8 Ethnicity: 34% African American, 24% Hispanic, 38% White Education: 41% < high school education Marital status: 39% married Times incarcerated*: M* = 15.4 Drug use: 57% injected drugs	Therapeutic Community Treatment – change in personal growth (Lifestyle Criminality Screening Form/Circumstances, Readiness and Suitability Scale) Recovery Perception Measure	Reincarceration—the first return to prison for either a technical violation or for a new offence (official records)	3 years	At 1 year following release, non-reincarcerated had significantly higher individual growth (*p* < 0.0003) and socialization (*p* < 0.001) Being over 36 (*p* < 0.04, OR 2.32), having TC treatment (*p* < 0.01 OR 0.44), reporting individual growth (*p* < 0.02, OR 1.41), and socialization (*p* < 0.01, OR 1.94) were predictive of non-incarceration 3 years following release	Good
*Dockery, 2019 New York, USA	Cohort study	*N* = 78 Age: M = 22 Ethnicity: 70% African American, 30% Latino 1^st^ offender: 39.8% Risk: 19.2% low risk, 26.9% high risk	Re-entry Programme (based on RNR) Positive/conflicted social support Risk	Recidivism	3 years	Being a first-time offender was only a significantly predictive factor of not reoffending (*p* < 0.01), 90% reduced odds of being arrested No impact of positive/conflicted social support on recidivism	Good
*Farrell, 2009 Chicago, Cleveland, and Houston, USA	Cohort study	*N* = 740 Age: *M* = 36.2 Chicago = 97.4% non-White Cleveland = 81.3% non-White Houston = 78.9% non-White 1/3 had spent time in segregation during incarceration	Mastery (9-point self-report scale) Self-esteem (Rosenberg’s Self-Esteem Scale) Religiosity (Fetzer Institute’s Multidimensional Measure of Religiousness and Spirituality)	Recidivism—self-reported arrest, self-reported drug use, official reincarceration	9 months	Mastery had no significant effect on re-arrest, controlling for re-entry problems Pre-release mastery was marginally significantly related to reincarceration (*p* = 0.080, OR = 5.75) but not when controlling for social stressors Mastery during incarceration did not have a significant effect on illegal drug use Self-esteem was not related to arrest, reincarceration, or illegal drug use A positive change in religiosity from pre to post release was associated with a marginally lower likelihood of reincarceration (OR = 0.665, *p* = 0.075). Change in religiosity (increase or decrease) was more likely to use illegal drugs in the follow-up than compared to those that reported no change (N.S)	Good
*Jensen, Williams, and Kane, 2020 Idaho, USA	Cohort study	*N* = 379	Therapeutic community Education—General Education Diploma Previous earnings, previous conviction, racial/ethnic origins, marital status, time served	Employment Mean quarterly earnings (post-release)	57 months	Participation in TC approached significance (*p* = 0.065) for post-release employment Being assigned to TC led to higher earnings post release (*p* < 0.0001). Previous earnings (*p* < 0.0001) and fewer historical convictions (*p* = 0.009) associated with higher post-release earnings	Good
Jung, 2014 Illinois, USA	Retrospective longitudinal cohort	*N* = 12,193 (6056 in Adult Transition Centre, 6136 in minimum security) Average sentence length—1 year Age: *M* = 31 7–14 months spent in ATC 81% Black 73% single	Adult Transition Centre (individual treatment, 35 ho primary programming, 23 days counselling)	Post-release earnings (official records) Employment	3 years	Quarterly earnings $82 higher for ATC (NS) In the first 2 years post-release earnings improved by $384 In the third year post-release earnings decline to $48 (NS)	Good
*Lattimore & Visher, 2013 USA	Quasi-experimental matched design	*N* = 1967 (wave 1), *N* = 984 (wave 2) Age: 28 30% White, 56% Black 12% homeless 64% employed 6 months prior to incarceration 75% family member also incarcerated 59% alcohol/drug use 2.76 years of incarceration 15 years old at first offence 41% person or violent crime	Prison re-entry services (Serious and Violent Offender Re-entry Initiative, SVORI) focussed on housing, health, education, employment, substance use, and criminal behaviour	Housing (self-reported) Employment (self-reported) Drug use (oral swab drug test) Criminal behaviour (administrative sources)	3 months	Housing: SVORI 35% more likely to report their name being on a lease/22% more likely to be living in own accommodation Employment: SVORI more likely to have formal pay for their current job (*p* = 0.001) with benefits (*p* = 0.028) Drug use: SVORI more likely to not have used marijuana in the last 30 days (*p* = 0.058) Criminal behaviour: SVORI more likely not to have committed any crimes since release (*p* = 0.033)	Good
*Link et al., 2019 USA	Cohort study	*N* = 1532 Age: 29.09 60% educated 53% Black 51% participated in SVORI 2.5 years average incarceration 35% substance abuse 61% prior employment 63% with antisocial peers	Mental and Physical Health (self-report) SVORI intervention	Barriers to reintegration (family conflict/employment status) Recidivism—self-reported crime from 9 questions Reincarceration—administrative data	15 months	1 SD increase in health limitations led to reduced odds of employment 3 months post release by 44% Depression had a significant effect on family conflict (*p* < 0.01) Employment showed direct negative effect on growth (*p* < 0.05) Financial problems at 9 months associated with increase in crime (*p* < 0.001) Depression and physical health limitations linked to crime and reincarceration through pathways involving family conflict, employment, and financial problems	Good
*Lugo et al., 2019 Ohio, USA	Quasi-experimental	*N* = 92,217	Unit management programmes: Thinking for Change, RFLS, Inside Out Dad, Cage Your Rage, Victim Awareness, Personal Responsibility of Violence and Money Smart	Return to prison (a new crime or parole violation)	3 years	Lower prevalence of prison return across all interventions (NS) Money Smart (*p* < 0.025), Victim Awareness (*p* < 0.025), and Cage Your Rage (*p* < 0.05) showed reduced crime or parole violation compared to control if started in the first year of incarceration	Good
Malouf et al., 2017 Mid-Atlantic, USA	Quasi- experimental	*N* = 40 (21 treatment, 19 TAU) 48% African American, 27% Caucasian, 10% other Age: 32 12 years of education	REVAMP (Re-entry values and mindfulness programme)	Mindfulness Self-control Substance misuse Recidivism (self-report/official records)	3 years	No significant differences between the two groups REVAMP group reported more criminal behaviour than TAU but NS TAU reoffended quicker than REVAMP (near significance)	Fair
*McDougall et al., 2013 UK	Quasi- experimental	*N* = 61 (25: MAPPA offenders in ADViSOR prison project/36: MAPPA offenders not in project) ADViSOR group with higher risk of harm Age: 39/44 Sentence length: 62 / 45 White: 100%/97%	ADViSOR behaviour monitoring whilst in prison	Recall/reoffending	1 year	ADViSOR negative prison behaviour predictive of recall (*p* < 0.001)	Good
McNeeley, 2018 Minnesota, USA	Randomised controlled trial	Phase 1 N = 244 (165 HRRR/79 control) Phase 2 N = 334 (146 HRRR/188 control) P1—fewer property crimes and fewer sex offenders Age: 36 (HRRR) / 34 (control) Sentence length: 71 months (HRRR)/74 months (control) HRRR—69% minorities, 75% high school diploma, 2.87 prior supervision failure Control—72% minorities, 79% high school diploma, 2.39 prior supervision failure	High Risk Revocation Reduction (HRRR) re-entry programme/standard case planning	Recidivism—re-arrest, reconviction, reincarceration for a new offence or revocation for a technical violation	69 months	Phase 1 = no significant differences in likelihood of any recidivism outcome Phase 2 = 76% control group vs. 62% HRRR re-arrested. Time to first re-arrest not significant HRRR participation associated with 23% lower risk of re-arrest (*p* = 0.074). No significant impact of HRRR on any recidivism outcome	Poor
Medlock, 2009 Oregon, USA	Randomized block design	*N* = 77 (38 treatment, 39 control) Age: 18–72	OPTIONS (employment counselling service)—3 months of 5 × weekly group sessions	Career Search Self-Efficacy Scale (self-report) Problem Solving Inventory (PSI) The Hope Scale (HOPE)	1 month	Significant difference on pre-post test results for treatment group for CSSE (*p* < 0.001), PSI (*p* = 0.03), and HOPE (*p* = 0.02), no differences between post-test and follow-up Significant difference between treatment and non-treatment at post-test for CSSE (*p* < 0.001, ES = 0.89) and follow-up (*p* < 0.001, ES = 0.98) Significant difference between treatment and non-treatment at post-test for HOPE (*p* < 0.05, ES = 0.59) and follow-up (*p* < 0.01, ES = 0.74)	Fair
Naser & La Vigne, 2006 Baltimore/Chicago, USA	Cohort study	*N* = 413 Age: *M* = 34 86% African American/Black, 4% White, 10% Other 23% married 62% with children	Family support (pre-release)—family support scale/family relationship support	Family support—family support scale/family relationship support	3 months	55% identified family as a factor that would be important in staying out of prison. 80% reported this at the post-release interview Prior to release, employment was the most important factor, but post-release housing was the most important factor (no statistical analysis)	Fair
O’Brien & Daffern, 2017 Australia	Cohort study	*N* = 82 Age: 20–67, *M* = 33 12 Aboriginal, 53 Caucasian, 17 other Minimum sentence: 34 months uhM = 3.3 previous sentences	Violence Reduction Programme—CBT-based group treatment targeting criminogenic needs (moderate and high intensity)	Violence Risk Scale Denial and Minimisation Checklist Victim Empathy Motivation Recidivism (police database)	3.65 years	Treatment completion = greater reductions in minimisation vs non-completers (*p* = 0.013, *d* = 0.73) Recidivism = VRS scores associated with increased violent recidivism (hazard ratio = 1.06, risk increase of 6%) VRS dynamic risk change scores not significantly correlated to violent reoffending, controlling for pre-existing risk Victim empathy change scores associated with violent recidivism (hazard ratio = 0.565) 43.5% decreased risk of violence Post-treatment motivation change in behaviour predicted violent recidivism (*p* = 0.058)	Fair
Russell et al., 2013 New Zealand	Qualitative longitudinal semi-structured interviews (pre and post-release)	*N* = 9 Age: 28–63 Sentence length: 12–126 months 100% sex offenders	Pre-release expectations on reintegration	Experience of reintegration	3–6 months	Pre-release: fear of the community reaction, need for pre-arranged accommodation, employment and social support, knowledge of probation officers and hope for a new life Post-release: negative community reactions stressful, accommodation issues, importance of getting a job and social support, probation officers unsupportive, building a new life and cultural accountability	18/20
*Semenza and Link, 2019 USA	Cohort study	*N* = 1697	SVORI re-entry programme Family support Years spent in prison Health insurance Drug use Race Education Marital status Reintegration barriers	Physical health Depression	15 months	1-unit increase in reintegration barriers is associated with 0.089 (*β* = 0.089, *p* < 0.01), 0.098 (*β* = 0.098, *p* < 0.05), and 0.083 (*β* = 0.083, *p* < 0.05) increases in physical health problems Reintegration barriers associated with depression at waves 2 and 3 (*p* < 0.01) Job at 3 months decreased physical health problems (*β* − 0.167, *p* < 0.05) Family support continued to show a protective effect on depression across the three time periods (*β* = − 0.141, *p* < 0.001; *β* = − 0.087, *p* < 0.001; *β* = − 0.180, *p* < 0.001)	Good
Simourd et al., 2016 Alaska, USA	Cohort study	*N* = 113 Age: 34 95% with at least 1 prior arrest 81.4% felony offences	Criminal Attitudes Programme (CAP) – CBT based group. 8–10 weeks, 2 x weekly	Criminal Sentiments Scale-Modified (CSS-M) Self-improvement orientation scheme (SOS-SR) Marlow-Crowne Social Desirability Scale (MCSDS) Recidivism	1 year	Lower CSSM at post-treatment (*p* < 0.001) Psychometric scores and recidivism did not reach significance—high change completers showed lower rates of recidivism (16.7%) than low change non-completers (35.1%), not a significant difference 8% decrease in re-arrest for each 1-point increase in attitude change, controlling for covariates	Fair
*Stansfield et al., 2017 USA	Cohort study	*N* = 1032	SVORI: re-entry programme Religious support	Substance use Criminal offending Employment	15 months	Religious support (*p* < 0.01), employment (*p* < 0.001), and SVORI participation (*p* < 0.01) were significantly related to substance abuse Religious support not related to criminal offending or employment	Good
*Visher et al., 2017 USA	Quasi-experimental study	*N* = 1697 Average age: 29 40% married 60% high school degree 2/3 employed prior to current incarceration + 50% Black 40% received drug and alcohol treatment Average 5 prior convictions Average age of first conviction: 16	SVORI: re-entry programme 12 individual pre-release service items	Time to re-arrest (during follow-up period) Number of arrests post-release	5 years	15% rearrested in 3 months, 82% arrested at 56 months SVORI programme approached significance for fewer post-release arrests (*p* = 0.08) Re-entry plan, personal relationship planning, criminal attitude training, anger management classes had beneficial significant effect on time to first re-arrest (*p* < 0.05) Re-entry cases, life skills, employment skills, mental health treatment had negative effects on time to first re-arrest (*p* < 0.05)	Good
*Wallace et al., 2016 USA	Quasi-experimental study	*N* = 550	SVORI re-entry programming Positive/negative familial support	Mental Health – SF12 Summary MH scale (MHS)	9 months	Family support in prison (positive or negative) had no effect on post-release MHS Negative family support post-prison impacted post-release MHS (*p* < 0.001). 1 SD increase in negative family support = 0.27 decrease in MHS	Good
Walters, 2016 USA	Cohort study	*N* = 951 Age: 19–84 (M = 35/SD = 9.65) 3–20 years of education 18.7% White, 68.9% Black, 11.6% Hispanic, 8% other 32% drug offences, 19.7% firearm violations, 18% supervised release violations/escape, 11% robbery, 3.8% fraud, 2.4% assault, 8.3% other crimes	Annual rates of incident reports received by an inmate (written by staff)	Recidivism—arrests, technical parole/supervised release violation leading to reincarceration (electronic files)	6 months	Age at release (*p* < 0.001), prior convictions (*p* < 0.001), and criminal thinking score (*p* = 0.023) significantly predict arrests for non-person crimes Age at release (*p* < 0.001), prior convictions (*p* < 0.001), and incident rates (*p* = 0.002) significantly predicted person crimes	Fair
Walters, 2020 USA	Cohort study	*N* = 1101, 724 in final analysis Average age = 34.64 (SD = 9.79) 11.33 years of education 19.6% White, 68.1% Black, 11.5% Hispanic, 0.4% Asian, 0.4% Native American	Psychological Inventory of Criminal Thinking (PICTS) – General Criminal Thinking Scale (GCT) Disciplinary reports (aggressive /non-aggressive)	First official arrest following release	12 months	Disciplinary infractions (p < 0.001) and GCT score (*p* < 0.001) associated with increased risk of reoffending Non-aggressive infractions (*p* < 0.001) and Reactive Criminal Thinking score (*p* < 0.001) predicted future offending	Fair

## Appendix 4

Table 3

**Table 3:** PRISMA statement checklist

Section and Topic	Item #	Checklist item	Location where item is reported
TITLE			
Title	1	Identify the report as a systematic review	Title Page
ABSTRACT			
Abstract	2	See the PRISMA 2020 for Abstracts checklist	Journal requirements of a 150-word abstract how well this can be met
INTRODUCTION			
Rationale	3	Describe the rationale for the review in the context of existing knowledge	P2/P3/P4 Manuscript
Objectives	4	Provide an explicit statement of the objective(s) or question(s) the review addresses	P4/ P5 Manuscript
METHODS			
Eligibility criteria	5	Specify the inclusion and exclusion criteria for the review and how studies were grouped for the syntheses	P6 (inclusion criteria) P7/8 (how studies were grouped) manuscript
Information sources	6	Specify all databases, registers, websites, organisations, reference lists and other sources searched or consulted to identify studies. Specify the date when each source was last searched or consulted	P5 (search strategy)
Search strategy	7	Present the full search strategies for all databases, registers and websites, including any filters and limits used	P5 (search strategry)
Selection process	8	Specify the methods used to decide whether a study met the inclusion criteria of the review, including how many reviewers screened each record and each report retrieved, whether they worked independently, and if applicable, details of automation tools used in the process	P7 (study selection and data) extraction
Data collection process	9	Specify the methods used to collect data from reports, including how many reviewers collected data from each report, whether they worked independently, any processes for obtaining or confirming data from study investigators, and if applicable, details of automation tools used in the process	P7 (study selection and data) extraction
Data items	10a	List and define all outcomes for which data were sought. Specify whether all results that were compatible with each outcome domain in each study were sought (e.g. for all measures, time points, analyses), and if not, the methods used to decide which results to collect	NA – any post-release outcome was included so no specific pre-determined outcomes. Information on likely outcomes given on p6 (inclusion/exclusion criteria)
	10b	List and define all other variables for which data were sought (e.g. participant and intervention characteristics, funding sources). Describe any assumptions made about any missing or unclear information	P7 (study selection and data extraction)
Study risk of bias assessment	11	Specify the methods used to assess risk of bias in the included studies, including details of the tool(s) used, how many reviewers assessed each study and whether they worked independently, and if applicable, details of automation tools used in the process	P7 (quality assessment(
Effect measures	12	Specify for each outcome the effect measure(s) (e.g. risk ratio, mean difference) used in the synthesis or presentation of results	NA – narrative synthesis and DAG
Synthesis methods	13a	Describe the processes used to decide which studies were eligible for each synthesis (e.g. tabulating the study intervention characteristics and comparing against the planned groups for each synthesis (item #5))	P7 (synthesis of research)
	13b	Describe any methods required to prepare the data for presentation or synthesis, such as handling of missing summary statistics, or data conversions	NA
	13c	Describe any methods used to tabulate or visually display results of individual studies and syntheses	Information on DAG visualisation given on p8
	13d	Describe any methods used to synthesize results and provide a rationale for the choice(s). If meta-analysis was performed, describe the model(s), method(s) to identify the presence and extent of statistical heterogeneity, and software package(s) used	P8 (synthesis of research)
	13e	Describe any methods used to explore possible causes of heterogeneity among study results (e.g. subgroup analysis, meta-regression)	NA
	13f	Describe any sensitivity analyses conducted to assess robustness of the synthesized results	NA
Reporting bias assessment	14	Describe any methods used to assess risk of bias due to missing results in a synthesis (arising from reporting biases)	NA
Certainty assessment	15	Describe any methods used to assess certainty (or confidence) in the body of evidence for an outcome	NA
RESULTS			
Study selection	16a	Describe the results of the search and selection process, from the number of records identified in the search to the number of studies included in the review, ideally using a flow diagram	Figure 1
	16b	Cite studies that might appear to meet the inclusion criteria, but which were excluded, and explain why they were excluded	Figure 1
Study characteristics	17	Cite each included study and present its characteristics	Appendix 3
Risk of bias in studies	18	Present assessments of risk of bias for each included study	Appendix 3
Results of individual studies	19	For all outcomes, present, for each study: (a) summary statistics for each group (where appropriate) and (b) an effect estimate and its precision (e.g. confidence/credible interval), ideally using structured tables or plots	Appendix 3
Results of syntheses	20a	For each synthesis, briefly summarise the characteristics and risk of bias among contributing studies	P10 (outcomes) – each sub-heading describes the studies contributing to the outcome and an overall comment about their quality
	20b	Present results of all statistical syntheses conducted. If meta-analysis was done, present for each the summary estimate and its precision (e.g. confidence/credible interval) and measures of statistical heterogeneity. If comparing groups, describe the direction of the effect	NA
	20c	Present results of all investigations of possible causes of heterogeneity among study results	NA
	20d	Present results of all sensitivity analyses conducted to assess the robustness of the synthesized results	NA
Reporting biases	21	Present assessments of risk of bias due to missing results (arising from reporting biases) for each synthesis assessed	NA
Certainty of evidence	22	Present assessments of certainty (or confidence) in the body of evidence for each outcome assessed	P10 – 12 synthesis of outcomes – end sentences
DISCUSSION			
Discussion	23a	Provide a general interpretation of the results in the context of other evidence	P17 – 21
	23b	Discuss any limitations of the evidence included in the review	P21
	23c	Discuss any limitations of the review processes used	P22
	23d	Discuss implications of the results for practice, policy, and future research	P23
OTHER INFORMATION			
Registration and protocol	24a	Provide registration information for the review, including register name and registration number, or state that the review was not registered	P5
	24b	Indicate where the review protocol can be accessed, or state that a protocol was not prepared	P5
	24c	Describe and explain any amendments to information provided at registration or in the protocol	P5
Support	25	Describe sources of financial or non-financial support for the review, and the role of the funders or sponsors in the review	P24
Competing interests	26	Declare any competing interests of review authors	Title page
Availability of data, code and other materials	27	Report which of the following are publicly available and where they can be found: template data collection forms; data extracted from included studies; data used for all analyses; analytic code; any other materials used in the review	Title page
